# The Effects of Nitrogen Fertilisation on the Anatomical Properties of the *Populus alba* L. Clone ‘Villafranca’ Juvenile Wood

**DOI:** 10.3390/biology11091348

**Published:** 2022-09-13

**Authors:** Iva Ištok, Nenad Potočić, Bogoslav Šefc, Tomislav Sedlar

**Affiliations:** 1 Faculty of Forestry and Wood Technology, University of Zagreb, 10000 Zagreb, Croatia; 2 Croatian Forest Research Institute, Division for Forest Ecology, 10450 Jastrebarsko, Croatia

**Keywords:** nitrogen fertilisation, *Populus alba* L. clone ‘Villafranca’, wood anatomical properties, juvenile wood, annual ring width

## Abstract

**Simple Summary:**

In 2020 and 2021, the world underwent the COVID-19 crisis, and in 2022, it is going through another war crisis. These crises have greatly affected the global economy and raw material supply chains, and trade is either disabled or very slow. The wood industry and other industries that depend on primary raw materials feel this effect strongly. These are important reasons why the wood industry should not depend on only one market and why it is necessary to study less valuable types of wood species as potential substitutes for the current ones, at least in products where this is possible due to their characteristics. Fertilising forested plantations with nitrogen fertilisers is not new, but its influence on different types of wood species has not been sufficiently investigated. Fast-growing clones are particularly interesting for such a procedure. This paper studies the effect of fertilisation with nitrogen fertilisers on the anatomical properties of the Poplar clone ‘Villafranca’.

**Abstract:**

This study investigates the effect of nitrogen fertilisation on the anatomical properties of the juvenile wood of the *Populus alba* L. clone ‘Villafranca’ from an experimental trial near the Drava River in Croatia. Nitrogen was applied for two consecutive years, and the immediate and potential post-treatment effects were investigated. The correlation between annual ring width (ARW) and individual wood anatomical properties was also examined. The fertilisation effect was confirmed after the first year of nitrogen application for all wood anatomical properties except the vessel lumen area (VLA). Fibre length (FL) was reduced, and double cell wall thickness (DCWT), ray area (RA), and cell wall area (CWA) increased. In contrast, the vessel lumen diameter (VLD) and vessel lumen area changed inconsistently between treatments. The second year of nitrogen application was determined to be effective for FL only. Due to the insignificant results in the second year of the application of nitrogen, the post-fertilisation effect of nitrogen fertilisation was not confirmed.

## 1. Introduction

Within the *Populus* genus, *Populus alba* L. is a unique pioneer species of riparian ecosystems [1]. It grows well on various site and soil conditions and is somewhat tolerant of climate changes, including drought, temperature, and salinity [2]. Among the *Populus alba* L. clones, ‘Villafranca’ is among the most represented and registered reference clones in different nursery trials [3,4]. It is mainly used for the afforestation of river lowlands and is planted in specialised plantations to produce furniture logs, pallets, etc. Previous research has determined its good [5] and confirmed above-average productivity and good survival [6]. Pelleri et al. [7] found that, after 9 to 10 years, *Populus alba* L. reaches a suitable diameter for commercial use (30 cm). 

In Croatia, along large rivers, there is a relatively large area of suitable habitats for growing poplars. Forests along the Drava River in north-western Croatia, including poplar forests, are very sparsely represented but are of great economic, ecological, and protective importance [8]. The same authors argue that preserving and breeding these forests is challenging and complex, while most pedosystematic units have insufficient plant nutrient properties. In addition to the numerous methods used in poplar plantation cultivation, fertilisation stimulates growth at various stages of tree development [9,10]. Field fertilisation trials are one of the most practical, reliable, and accurate ways of determining the site’s need for nutrients [11]. 

In the past, fertilisation became a potentially important silvicultural method. Each plant species generally requires a certain amount of nutrients to ensure its optimal growth. Fertilisation efficiency (the percentage of the applied element assimilated by the target trees) varies with the chemical form of the element and the timing of application. Weeds must also be controlled for seedlings to maximise fertiliser utilisation [12]. The lack of water and nutrients can be solved through the control of weed vegetation and the lack of nutrients through fertilisation. Weed control without irrigation increases poplar growth, while irrigation without weed control does not produce this effect [13]. Moreover, clonal variability in response to soil fertility and fertilisation is a large area of uncertainty. Physiological characteristics and how different poplar clones develop are of the greatest importance for the parameters of the size and quality of the yield. Short rotations carry the enhanced risk of nutrient depletion in the soil due to the frequent removal of juvenile wood rich in nutrients [14,15]. Fertilisation, given at the right time and in agreement with other silvicultural measures, can not only increase growth but can also increase the value of the yield by increasing the proportion of wood suitable for peeling and other more valuable products. Large quantities can be applied but do not contribute to further growth [16]. Nitrogen is the most limiting element for poplar growth [17] and generally causes the most significant changes in wood properties [18]. Poplar’s high nutrient demands are associated with short rotations in plantations and their expected high productivity [17]. Such silvicultural practices often lead to nutrient depletion from already depleted sites [19]. This has resulted in the increased application of multiple fertiliser doses in plantation cultivation. 

Poplars are usually grown in short rotations resulting in a high proportion of juvenile wood. Wood is naturally a variable material because it is a product of the living tree metabolism. The result is the pronounced variability of its properties due to tree physiology, external influences on its growth, and tree age. Increased tree increments due to fertilisation may be associated with a more significant change in wood properties than other cultivation methods [20].

Primarily, research on the effect of fertilisation has focused on increasing the diameter and height increment of poplar trees [21,22,23,24,25]. The effect of nitrogen fertilisation on poplar wood structure has been the subject of a certain number of investigations, from the early years to the present [26,27,28,29]. However, the available data are inconsistent and contrasting. All these studies indicate that the effect of nitrogen varies between poplar species, and there is no predictable response to nitrogen fertilisation [30]. The use of fertilisation in forestry is constantly increasing and requires more methods to identify the actual needs for proper fertiliser doses. Furthermore, it is essential to investigate the effect of nitrogen fertilisation treatments on wood quality in more detail, emphasising its duration and magnitude.

The wood anatomical properties of *Populus alba* L. from the natural population on the same site along the Drava River were previously investigated [31]. The current research is the continuation of efforts to determine the potential of *Populus alba* L. breeding and utilisation.

The aim of this study is:

(a) To determine the selected wood anatomical properties of the juvenile wood of the *Populus alba* L. ‘Villafranca’ clone from an experimental fertilisation trial.
(b) To investigate the effect of different nitrogen fertiliser doses on the juvenile wood of selected wood anatomical properties of the *Populus alba* L. ‘Villafranca’ clone during treatment and post-treatment.
(c) To investigate the correlation between annual ring width and wood anatomical properties following nitrogen fertilisation.

## 2. Materials and Methods

### 2.1. Site and Study Materials

In the Forest Office Varaždin, Croatia, Management Unit “Varaždinske podravske šume”, Department 3a (46°23′ N, 16°06′ E), an experimental trial of different poplar clones was set up in 2001, including one of the *Populus alba* ‘Villafranca’. The experiment was established as a randomised block system with four replicates and ten clones by planting 1/1 seedlings to a depth of 80–100 cm. Eighty seedlings per clone were planted at 4.25 × 4.25 m spacing. The total area of the experiment was 1.44 ha, while each area was divided into monoclonal plots of about 0.144 ha for each clone. From each of the four monoclonal plots (0.036 ha), four trees were selected for harvesting. These are deep alluvial carbonate clay loam to sandy loam soils of high productive capacity due to a favourable mechanical system, high water capacity and nutritional potential, and where groundwater in the vegetation minimum is inaccessible to the roots of major forest species. The mean annual temperature is 10.8 °C, and the total annual precipitation is 728 mm [32]. In the MU “Varaždinske podravske šume”, soils with high water permeability and poor water resistance predominate, which is why the existing ecosystems, mostly willow and poplar forests and forest plantations, are easily susceptible to damage. In 2004 and 2005, a fertilisation experiment was incorporated into the existing trial to test the response of clones to four nitrogen fertiliser doses: 0, 100, 200, and 300 kg N/ha. In 2004, fertilisation began with the following treatments: N0—N0P1K1 (control); N1—N1P1K1; N2—N2P1K;1 and N3—N3P1K1, where the concentrations were N1 = 100 kg/ha, N2 = 200 kg/ha, N3 = 300 kg/h, P1 = 100 kg/ha P_2_O_5_, and K1 = 100 kg/ha K_2_O. Treatments differed in the amount of nitrogen added. The same treatment was carried out in 2005. In both years, fertilisation was performed using two equal doses in two applications, at the end of April and at the end of May.

### 2.2. Sample Preparation

Sixteen trees of the ‘Villafranca’ clone were collected: four control trees (N0) and twelve trees with different nitrogen applications (N1, N2, and N3), one from each of four replicates within the trial. Five-centimetre-thick disks were cut from each tree at breast height (1.3 m). Radial segments (north-south orientation) were cut from each disk. Four annual growth rings were selected and marked along one radius: two related to 2004 and 2005 (during fertilisation) and two related to 2006 and 2009 (after fertilisation).

### 2.3. Measurement of Fibre Length

The maceration procedure was performed using Franklin’s reagent [33] to separate fibres for measuring fibre length. Small wood chips from each previously selected annual ring were cut and placed in test tubes. An acetic acid and hydrogen peroxide mixture (1:1) was poured into the test tubes and placed in an oven for 48 h at 60 °C. The macerated material was stained and mounted on microscope slides in glycerine. To determine fibre length, 40 unbroken fibres were measured using a light microscope (Carl Zeiss Jena, Carl-Zeiss-Promenade 10, 07745 Jena, Germany), digital camera (Dino-Lite), and DinoCapture 2.0 software (Dino-Lite Europe, 1321 NN Almere, The Netherlands).

### 2.4. Measurement of Annual Ring Width

Radial segments (north-south orientation) were dried and sanded. Then, an annual ring width measurement was performed on a Lintab 6 (Rinntech-Metriwerk GmbH & Co. KG Hardtstr. 20-22 D-69124 Heidelberg, Germany) measuring table with a precision of 1/100 mm. Both radii were measured, and mean values were calculated.

### 2.5. Measurement of Other Wood Anatomical Properties

Each selected annual growth ring was cut in the form of sample blocks sized 10 (tangential) × 10 (radial) × 20 (longitudinal) mm. The sample preparation included softening and cutting into transverse and tangential sections (30 µm) using a sliding micro-tome. The sections were stained with a mixture of safranin and Astra blue, washed in a 70% and 96% ethanol solution, and mounted on microscope slides using Euparal. Using an Axio Zoom.V16 stereo microscope and AxioVision software (Carl Zeiss) connected to a digital camera (5 MP), five images as random replicates were taken in the transverse section: one from the earlywood region, one from the latewood region, and three from the middle part of the annual growth ring [34]. Likewise, three images were taken in the tangential section. Due to the complexity of measuring the ray area by the individual manual marking of each ray in the software, a statistical analysis confirmed no significant differences between the use of three and five images per section. The samples were photographed at ×160 (fibre lumen) and ×100 (vessel lumen and ray) magnification. Fibre lumen diameter (FLD) (µm), vessel lumen diameter (VLA) (µm), and ray area (RA) (%) measurements were performed using ImageJ software (ImageJ 2014). The ray area was calculated as the ratio between the ray total area (µm^2^) and the image area (µm^2^) multiplied by 100. The fibre lumen area (FLA) (%) was calculated as the ratio between the fibre total lumen area (µm^2^) and the image area (µm^2^) divided by 100, while the vessel lumen area (VLA) (%) was calculated as the ratio between the vessel total lumen area (µm^2^) and the image area (µm^2^) multiplied by 100. The double cell wall thickness (DCWT) was measured directly on a light microscope using an objective with a microscope scale and ×960 magnification. The cell wall area (CWA) (%) is the remaining percentage from the subtraction of the vessel lumen area (%), fibre lumen area (%), and ray area (%) from the unit area.

### 2.6. Statistical Analysis

Statistical analysis of all the results was carried out in Statistica 14.0 (TIBCO Soft Inc., 3307 Hillview Avenue Palo Alto, CA 94304, USA). Repeated measures analysis of variance and univariate variance analysis (ANOVA) were used to test the effect of the treatments, years, and the years and treatment interaction. Statistical significance was determined by *p*-value. If the *p*-value is less than or equal to 0.05, the null hypothesis is rejected, and the differences are declared statistically significant. In contrast (*p* ≥ 0.05), the null hypothesis is not rejected, and the differences are not statistically significant. In the second stage of the variance analysis, the univariate variance analysis model, a post hoc test (Tukey test) of multiple comparisons of individual treatment pairs, was used. The differences are declared statistically significant if the *p*-value is less than or equal to 0.05. Finally, a correlation analysis was performed to investigate the relationship between the ARW and individual wood anatomical properties over the years.

## 3. Results

### 3.1. Wood Anatomical Analysis and Effect of Treatment over Years

**Figure 1:**
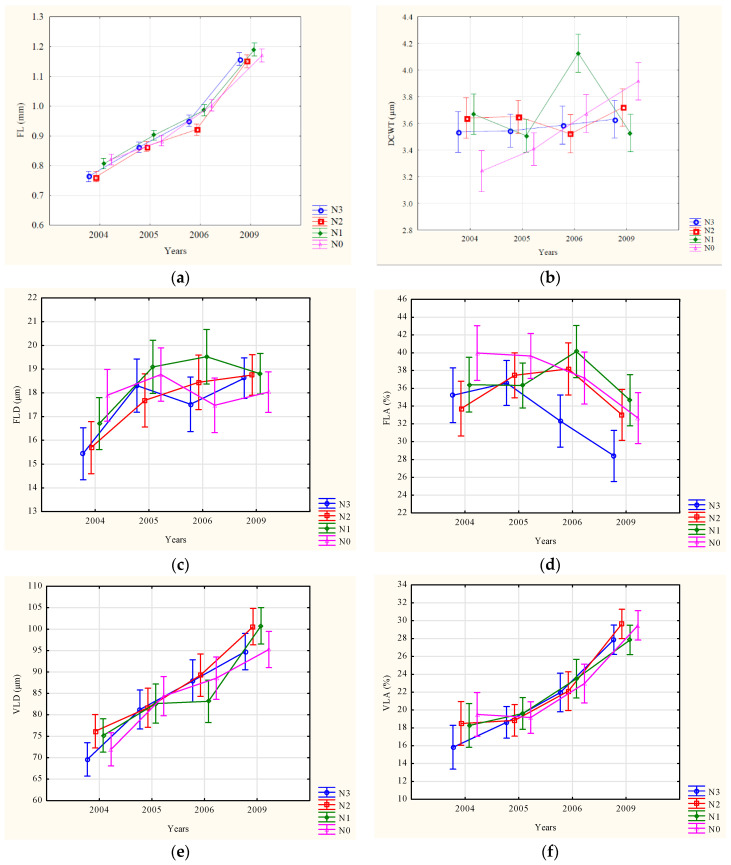

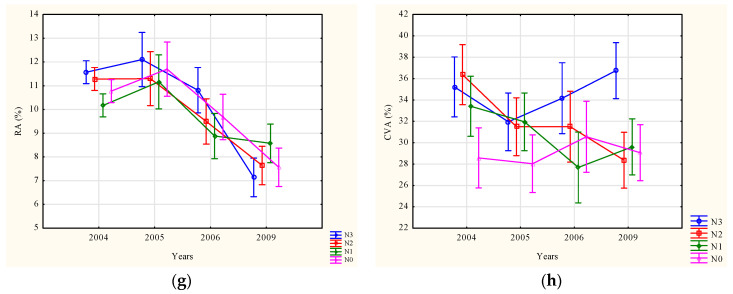
Trends of the anatomical properties of juvenile wood for control and different levels of nitrogen fertilisation by year. (**a**) FL—fibre length; (**b**) DCWT—double cell wall thickness; (**c**) FLD—fibre lumen diameter; (**d**) FLA—fibre lumen area; (**e**) VLD—vessel lumen diameter; (**f**) VLA—vessel lumen area; (**g**) RA—ray area; (**h**) CWA—cell wall area. The vertical bars represent 0.95 confidence intervals.

Mean values and the standard deviation for all investigated wood anatomical properties are given in Table 1. Trends for control and different levels of nitrogen fertilisation by year within juvenile wood are presented in Figure 1. 

The lowest average FL was after treatments N2 and N3 in 2004 (0.76 mm). In the last year measured (2009), the average FL was highest after all treatments (1.17 mm after N0, 1.19 mm after N1, 1.15 mm after N2, and 1.16 mm after N3) (Table 1). The average DCWT was lowest after treatment N0 in 2004 (3.24 µm) but highest after the same treatment in 2009 (3.91 µm). The highest average DCWT was reached after treatment N2 in 2005 (3.65 µm), while the total highest value was in 2006 (4.13 µm). The highest average DCWT was reached after treatment N2 in 2005 (3.65 µm), while the total highest value was in 2006 (4.13 µm). The highest average FLD and FLA were after treatment N1 in 2006 (19.52 µm and 40.14%). Treatment N3 had a similar effect on FLD and FLA, resulting in the total lowest values for FLD in 2004 (15.43 µm) and FLA in 2009 (28.41%).

Vessel characteristics also varied with treatments between the years (Table 1). The average VLD was highest after treatment N2 in 2004 (76.17 µm) and 2006 (89.27 µm). The highest VLD was after treatment N0 in 2005 (84.36 µm), while it was highest after treatment N1 in 2009 (100.77 µm). Average VLD showed the lowest values after treatment N3 in 2004 (69.62 µm), 2005 (81.25 µm), and 2009 (94.77 µm) and after treatment N1 in 2006 (94.77 µm). Similarly, average VLA showed the lowest values after treatment N3 in 2004 (15.83%), 2005 (18.62%), and 2006 (21.96%). For both VLD and VLA, treatment N1 resulted in the lowest values in 2006 (83.16 µm) and 2009 (27.85%). Fertilisation with the highest nitrogen dose (treatment N3) decreased the fibre and vessel characteristics compared to the control (N0). However, the highest nitrogen dose (treatment N3) increased RA and CWA (Table 1). The control trees (N0) showed lower values of the two properties in comparison to those fertilised. Average RA was highest after treatment N3 in 2004 (11.57%), 2005 (12.10%), and 2006 (10.81%), but lowest after the same treatment in 2009 (7.14%). Average CWA was highest after treatment N2 in 2004 (36.37%) and after treatment N3 in 2006 (34.17%) and 2009 (36.75%). The exact values of CWA were determined after treatments N1 and N3 in 2005 (31.95%) (Table 1). The lowest average CWA was after treatment N1 in 2006 (27.68%).

The effect of years was highly significant for all wood anatomical properties except CWA. However, the interest was identifying the influence of different nitrogen fertilisation treatments during selected years. The results and discussion will focus on the treatment’s main effect and the years-by-treatment interaction terms.

In years during fertilisation and after fertilisation, the years-by-treatment interaction was significant for all fibre characteristics (FL, DCWT, FLD, and FLA), with DCWT being highly significant (Table 2). Significant interaction was determined for RA and CWA as well. Conversely, the same interaction was insignificant for vessel characteristics (VLD and VLA).

After all the treatments, an increasing radial trend was determined for FL (Figure 1). A similar trend with increasing values between the years was detected for VLD and VLA. On the other hand, RA showed a decreasing trend from 2004 to 2009 after all treatments. Compared to the described trends, DCWT, FLD, FLA, and CWA showed no consistent radial pattern after different treatments over the years (Figure 1).

### 3.2. Differences in Wood Anatomical Properties between Treatments

The results of the ANOVA with Tukey post hoc test for the first two years (2004 and 2005) are given in Table 3, and the results for years after fertilisation (2006 and 2009) are given in Table 4.

The main effect of the treatment was significant for FL only in both years of fertilisation (Table 3). For both years, significance was determined between N1 and N2, and between N1 and N3. DCWT, FLD, FLA, RA, and CWA were significantly affected by the treatments in the first year only. On the other hand, the addition of nitrogen fertiliser had no significant difference in VLD and VLA in 2004 and 2005. Most wood anatomical properties show a significant difference between treatments N0 and N2, N0 and N3, N1 and N2, as well as between N1 and N3 in 2004, and between treatments N0 and N2 in 2005 (Table 3). The difference between N0 and N1 was determined only in DCWT.

The main effect of the treatment was significant for DCWT and FLA only in both years after fertilisation (Table 4). The first year after fertilisation showed a significant difference between treatments in FLD and RA, while in 2009 this was detected in CWA only. Most wood anatomical properties show a significant difference between treatments N0 and N3, N1 and N2, as well as between N1 and N3 in 2006, and between treatments N0 and N3, and N1 and N3 in 2005 (Table 3). The difference between the control (N0) and the lowest nitrogen dose (N1) was determined only in DCWT, as in the two years during fertilisation. When observing all wood anatomical properties, the highest nitrogen treatment (N3) mainly differed from treatments N0 and N1.

### 3.3. Growth Rate and Wood Anatomical Properties

The mean values and standard deviations of annual ring widths are given in Table 5.

From the results in Table 5, Wider ARW are detected in two years of fertilisation for all treatments, including N0. Furthermore, differences in ARW between the years following each treatment are statistically significant.

In the two years during and two years after fertilisation, FL, VLD, and VLA were strongly negatively correlated with ARW following all treatments (Table 6). However, RA was strongly positively correlated with ARW. DCWT was weakly negatively correlated with ARW following treatments N1 and N2 but strongly negatively correlated following treatments N0 and N3. FLA showed different degrees of correlation with ARW, all being positive. A moderately positive correlation was detected between FLD and ARW after treatment N0, while other treatments resulted in a moderately negative correlation. While CWA moderately correlated with ARW following all treatments, it was positively correlated after N1 and N2 and negatively after N0 and N3.

## 4. Discussion

Nowadays, fertilisation experiments are more often performed on softwoods than hardwoods, which means that more literature findings on this topic relate to softwoods.

In some cases, the impact of the application of one fertiliser on tree productivity is observed even after several years [35]. However, the short-term effect of fertilisation on the anatomical properties of wood has been confirmed [36]. Consequently, such effects depend on the treatment used (type of nutrients, dose, etc.), as well as on the genotype of the trees [28,37,38].

Nitrogen fertilisation on the clone ‘Villafranca’ was carried out in two consecutive years, 2004 and 2005. In order to investigate the immediate and possible post-treatment effect of nitrogen fertilisation on wood anatomical properties, two years during fertilisation (2004 and 2005) and two years after fertilisation (2006 and 2009) were included in the measurements. Data on radial variations in the wood anatomical properties of treated trees are also very scarce. Since the null hypothesis was rejected for specific wood anatomical properties, especially with the results for 2005, comparisons were made between each pair of treatments. 

Studies exist on the influence of fertilisation on wood properties. However, they are sometimes difficult to interpret because the response to fertilisation depends on the age of the trees, the composition of the fertiliser, and the period in which the fertilisation is carried out [20]. However, since external environmental factors provide the physical conditions for all biological processes, fertilisation affects the tree’s growth and thus, in a certain way, the wood structure [39].

The results of this research indicate that only FL was significantly affected by different nitrogen treatments during both years of fertilisation. Research on the *Populus deltoides* clone [40] reported longer, thicker, wider fibres and wider vessels after three years of fertilisation. In this research, the application of nitrogen reduced FL in 2004, while only N1 increased FL in 2005. This is supported by a few sources on different wood species [41,42]. The shorter fibre result is possibly due to decreased fusiform cambial initials following different environmental conditions [26]. DCWT increased after all three nitrogen doses. Higher nitrogen doses (N2 and N3) had a greater effect on individual wood anatomical properties. 

The larger dimensions of the fibres and vessels after the addition of nitrogen are possible evidence of the maturity of cambium [40]. FLD and FLA decreased after the medium (N2) nitrogen dose in 2004. Only VLA showed no significant effect of treatments during fertilisation. Nitrogen fertilisation on *Populus spp.* resulted in shorter fibres, but in contrast, thinner cell walls, wider vessels and fibres, and a decrease in the cell wall area [29,30,38]. All these results indicate that the nitrogen effect varies with different poplar species. RA also increased after higher doses (N2 and N3) were applied. Early findings on hardwood support this result [20].

The first application (first year) of nitrogen fertiliser was expected to affect its structure. The observed years significantly differed in the amount of precipitation within the growing season. There was an average amount of precipitation in 2004, while the humid year of 2005 is particularly noteworthy. Average temperatures but typical of the extremely high amounts of precipitation in 2005 could explain the small or insignificant effect of fertilisation in that year. We also have to consider the possibility that part of the fertiliser was not used but washed away on such light soils. However, the differences in FL in 2005 were not detected in comparison to the control trees (N0), but only between the nitrogen treatments. This could be due to the differences in the microenvironment of the trees [43], despite the treatment applied. Environmental factors constantly interact in a complex manner, complicating the analysis of the effects of the selected factors [39].

The clone ‘Villafranca’ trees were harvested young, producing a high proportion of juvenile wood. Juvenile wood is considered to be highly variable in properties [26]. This could explain the radial trends in the certain wood anatomical properties investigated. In juvenile wood, fibre length usually increases with age [44]. FL showed an increasing trend as the age of the clone ‘Villafranca’ increased. This suggests that cambial age has an essential effect on the length of wood fibres [43]. The application of nitrogen fertiliser resulted in an increase in VLD and VLA with age as well. While the years-by-treatment interaction was significant for FL, similar trends in VLD and VLA caused it to be insignificant. Unlike other wood anatomical properties, only RA showed a consistent decrease with age. Concerning the significant interaction effect, other fibre characteristics (DCWT, FLD, and FLA) display non-consistent radial patterns with treatments. The cell wall thickness of juvenile wood is expected to increase with age and is confirmed after fertilisation in conifers [45,46,47]. This trend was seen only after the N0 treatment. According to the results, the change in CVA could be controlled by the complex interaction of the parameters included in its calculation. In *Eucalyptus grandis* fertilised wood, significant differences have been determined in fibre length, fibre diameter, fibre wall thickness, and vessel diameter following the radial position [48].

The short-term effect of nitrogen on the anatomical properties of poplar wood has been confirmed in the literature [38,40] and in this research. The long-term response to growth was detected after intensive and repeated nitrogen fertilisation [49]. Although fertilisation can improve growth and affect wood properties, its effect on tree growth is not long lasting and requires repeated fertilisation treatments [26]. While nitrogen fertilisation hardly affected the anatomical properties of the wood in 2005, significant differences were detected two years after fertilisation. VLD, VLA, and CWA showed no significant effect post fertilisation, while the same was true in 2005. Consequently, no differences were expected after two years of treatment. Insignificant differences between treatments were determined for DCWT, FLD, FLA, and RA in 2005. However, they were calculated as significant the year after. No such effect was reported in the literature. Inconsistent data resulted from the analysis of individual wood anatomical properties during fertilisation and post fertilisation. Compared to the control (N0), the effect was significant after three years (in 2006) for FL and FLD. DCWT was significantly different between treatments in 2006, but not in 2005. The effect of treatment after 2006 was determined only for DCWT and CWA.

Usually, the response to treatment is found in the year following fertilisation or in the year of re-fertilisation [49]. Significant effects up to four years might be expected [36] but in continuation over the years. The results point to the possibility of a prolonged effect of nitrogen fertilisation despite the suggestion that the site may not have been in a condition to respond to fertilisation in 2005. Some preliminary data confirm the excellent response of this clone to nitrogen fertilisation. However, it is not easy to make definite conclusions about the long-term results of this research because of certain confounding influences, such as overall site and climatic conditions in the years investigated.

Wood quality is assumed to deteriorate, and the growth rate increases with the intensity of the silvicultural treatments applied [50], such as with fertilisation. However, it is challenging to characterise the effect of nitrogen fertilisation as exclusively positive or negative. This is due to the different requirements regarding the anatomical properties of poplar wood for specific purposes. On the one hand, the paper industry requires longer wood fibres with thinner walls and thus larger diameters and proportions of their lumens [47,51]. On the other hand, thicker fibre walls and a more significant proportion of wood cell walls contribute to increased wood density and thus better mechanical properties [43]. The results of this research could be interpreted accordingly. Nitrogen fertilisation reduced FL to some extent but increased DCWT and CWA. This effect is likely to be considered positive regarding the different poplar wood utilisation. 

Nitrogen fertilisation increases the annual ring widths of different species [27,49]. The faster growth rate in 2004 and 2005 was associated with younger tree age and response to nitrogen fertilisation. The nitrogen quantities required for sustainable growth of different *Populus* species are between 100 and 300 kg/ha/year [17]. The amounts in this range were applied in the current research. Good growth, especially in 2004, can be attributed to the synergistic influence of initial soil fertility, fertilisation, and favourable climatic conditions, which affected the favourable state of nutrition. 

The variation in DCWT was poorly explained by ARW following the lowest and medium nitrogen dose (N1 and N2). This was supported by findings on the positive effect of the treatment on ARW, an important factor explaining the effects on primarily cell wall thickness [47,52]. A similar correlation was detected for FLA after the N1 treatment. Other wood anatomical properties were moderately or strongly correlated with ARW, both positively and negatively. This suggests that the observed changes in wood anatomical properties are associated with growth responses [41]. ARW is often weakly correlated with wood density in fertilised trees [36,52]. CWA and wood density are dependent parameters within annual growth rings. However, this research displays the opposite correlation between CWA and ARW.

## 5. Conclusions

Only FL was significantly affected by the different nitrogen treatments during the application of nitrogen in two consecutive years. Each of the three levels of nitrogen fertilisation differently affected changes in the anatomical properties of the juvenile wood of the clone ‘Villafranca’ in each year of application. In the first year, fertilisation reduced FL and increased DCWT, RA, and CWA, while VLD and VLA were not significantly affected. Inconsistent changes were detected in FLD and FLA. 

While most wood anatomical properties were not affected by the treatments in 2005, the short-term effect of nitrogen fertilisation is seen in the results in 2004. The post-treatment effect was detected in FL, DCWT, FLD, FLA, and RA. However, due to the insignificant results in 2005, it is impossible to confirm the post-treatment effect of nitrogen fertilisation on these properties.

In comparison to the control (N0), most wood anatomical properties were changed after the medium (N2) and highest nitrogen dose (N3).

The most significant source of variation was determined to be the treatment in the first year of fertilisation. The effect of the years and time interaction significantly affected most of the wood anatomical properties investigated.

Both positive and negative effects of nitrogen fertilisation on wood anatomical properties regarding different demands for wood utilisation can be deduced from this research. A moderate to strong correlation between individual wood anatomical properties and ARW following nitrogen fertilisation was determined, both positive and negative. Furthermore, response to growth was detected in all wood anatomical properties except DCWT and FLA.

The results of this research may contribute to a better understanding of the effects of nitrogen fertilisation on the quality of poplar juvenile wood, providing essential data for the future use of fertilisers in poplar breeding. The research can also serve as a basis to support and increase the ecological and economic value of Drava floodplain forests near Varaždin.

## Figures and Tables

**Table 1:** Descriptive statistics for different years of the anatomical properties of the juvenile wood of the clone ‘Villafranca’. Measurements per group of each treatment in a year are *n* = 160 (fibre length), *n* = 120 (double cell wall thickness), *n* = 20 (fibre lumen diameter, vessel lumen diameter, and vessel lumen area), and *n* = 12 (fibre lumen area, ray area, and cell wall area).

Property	Year	Treatment									
		Σ		N0		N1		N2		N3	
		Mean	SD	Mean	SD	Mean	SD	Mean	SD	Mean	SD
FLA (mm)	2004	0.79	0.11	0.82	0.12	0.81	0.10	0.76	0.10	0.76	0.12
	2005	0.88	0.11	0.88	0.10	0.90	0.10	0.87	0.12	0.86	0.10
	2006	0.96	0.13	1.00	0.11	0.99	0.11	0.92	0.14	0.95	0.12
	2009	1.17	0.14	1.17	0.11	1.19	0.15	1.15	0.15	1.16	0.14
DCWT (µm)	2004	3.52	0.85	3.24	0.67	3.67	1.17	3.64	0.63	3.53	0.83
	2005	3.55	0.70	3.41	0.47	3.51	0.83	3.65	0.66	3.54	0.74
	2006	3.77	0.84	3.67	0.68	4.13	1.03	3.52	0.79	3.58	0.62
	2009	3.67	0.79	3.91	0.63	3.52	0.64	3.72	0.83	3.63	0.98
FLD (µm)	2004	16.45	2.57	17.90	2.14	16.70	2.24	15.69	1.73	15.43	3.40
	2005	18.53	2.50	18.77	2.66	19.10	1.57	17.68	1.99	18.30	3.45
	2006	18.30	2.62	17.47	2.55	19.52	2.53	18.45	3.01	17.52	2.17
	2009	18.49	1.89	18.03	2.16	18.80	1.63	18.75	1.64	18.62	2.17
FLA (%)	2004	36.29	5.47	39.97	4.74	36.41	7.10	33.71	4.90	35.21	3.87
	2005	37.31	4.37	39.64	5.09	36.32	3.74	37.46	3.16	36.60	5.08
	2006	37.02	5.52	37.15	5.90	40.14	3.78	38.18	4.46	32.32	5.70
	2009	31.91	5.26	32.64	4.62	34.65	5.36	33.02	2.63	28.41	6.34
VLD (µm)	2004	73.31	8.99	71.95	7.51	75.19	8.62	76.17	8.66	69.62	10.03
	2005	82.63	9.93	84.36	9.57	82.64	10.79	81.67	11.04	81.25	9.38
	2006	86.91	11.15	88.55	11.83	83.16	12.30	89.27	10.61	87.93	9.46
	2009	98.05	9.78	95.25	7.69	100.77	11.39	100.58	9.36	94.77	9.31
VLA (%)	2004	17.81	5.57	19.50	3.92	18.27	7.88	18.49	4.61	15.83	4.77
	2005	18.81	3.94	19.15	4.21	19.61	3.68	18.84	3.71	18.62	4.27
	2006	22.37	4.85	22.97	3.90	23.51	4.48	22.11	5.12	21.96	5.76
	2009	28.72	3.66	29.48	3.20	27.85	3.95	29.64	4.28	27.87	3.21
RA (%)	2004	10.98	0.96	10.78	0.69	10.17	0.67	11.28	1.08	11.57	0.82
	2005	11.64	1.90	11.70	2.40	11.16	0.91	11.30	1.21	12.10	2.70
	2006	9.78	1.71	9.68	1.11	8.88	1.19	9.50	1.06	10.81	2.65
	2009	7.76	1.41	7.56	0.72	8.57	2.03	7.64	0.92	7.14	1.52
CWA (%)	2004	33.76	5.58	28.57	3.71	33.41	5.41	36.37	5.01	35.22	5.02
	2005	31.27	4.93	28.03	5.36	31.95	5.08	31.50	3.93	31.95	4.05
	2006	31.05	5.89	30.56	5.71	27.68	4.33	31.50	7.54	34.17	4.70
	2009	31.25	5.52	29.06	2.80	29.60	3.80	28.36	2.54	36.75	7.23

Notes: FL—fibre length; DCWT—double cell wall thickness; FLD—fibre lumen diameter; FLA—fibre lumen area; VLD—vessel lumen diameter; VLA—vessel lumen area; RA—ray area; CWA—cell wall area; SD—standard deviation.

**Table 2:** Results of repeated measures analysis of variance ANOVA for the anatomical properties of the juvenile wood of the clone ‘Villafranca’ in different years and treatments by year.

Property	Source	SS	d.f.	MS	F	*p*
FL (mm)	Years	50.41	3	16.80	1364.30	0.000000
	Years × treatment	0.272	9	0.030	2.50	0.008859
DCWT (µm)	Years	17.15	3	5.72	9.81	0.000002
	Years × treatment	47.60	9	5.29	9.07	0.000000
FLD (µm)	Years	241.2	3	80.40	17.68	0.000000
	Years × treatment	107.9	9	12.00	2.63	0.006425
FLA (%)	Years	845.0	3	281.70	11.82	0.000001
	Years × treatment	477.7	9	53.10	2.29	0.023917
VLD (µm)	Years	25165	3	8388	93.10	0.000000
	Years × treatment	1256	9	140.00	1.55	0.131871
VLA (%)	Years	5591.0	3	1863.70	89.34	0.000000
	Years × treatment	119.9	9	13.30	0.64	0.763408
RA (%)	Years	412.99	3	137.66	97.23	0.000000
	Years × treatment	43.86	9	4.87	3.44	0.000773
CWA (%)	Years	219.6	3	73.20	2.72	0.047344
	Years × treatment	593.5	9	65.90	2.45	0.013121

Notes: FL—fibre length; DCWT—double cell wall thickness; FLD—fibre lumen diameter; FLA—fibre lumen area; VLD—vessel lumen diameter; VLA—vessel lumen area; RA—ray area; CWA—cell wall area; SS—sum of squares; d.f.—degrees of freedom; MS—mean sum of squares.

**Table 3:** Results of repeated measures analysis of variance ANOVA for the anatomical properties of the juvenile wood of the clone ‘Villafranca’ in two years (2004 and 2005) during nitrogen fertilisation.

Property	Year	Source	SS	d.f.	MS	F	*p*	Tukey Post Hoc *
FL (mm)	2004	Treatment	0.41983	3	0.1399	11.2	0.000000	N0-N2/N3, N1-N2/N3
	2005		0.1646	3	0.0549	4.63	0.003253	N1-N2, N1-N3
DCWT (µm)	2004	Treatment	13.6881	3	4.5627	6.31	0.000334	N0-N1, N0-N2, N0-N3
	2005		0.0000	3	1.2073	2.57	0.053922	N0-N2
FLD (µm)	2004	Treatment	75.2637	3	25.0879	4.15	0.008825	N0-N2, N0-N3
	2005		22.6017	3	7.5339	1.18	0.321366	
FLA (%)	2004	Treatment	256.4162	3	85.4721	3.05	0.038165	N0-N2
	2005		81.1634	3	27.0545	1.43	0.246736	
VLD (µm)	2004	Treatment	543.5537	3	181.1846	2.37	0.077484	
	2005		114.6873	3	38.2291	0.37	0.777731	
VLA (%)	2004	Treatment	145.6191	3	48.5397	1.60	0.196552	
	2005		11.0784	3	3.6928	0.23	0.872841	
RA (%)	2004	Treatment	13.6097	3	4.5366	6.56	0.000918	N1-N2, N1-N3
	2005		6.4606	3	2.1535	0.56	0.643333	
CWA (%)	2004	Treatment	425.2000	3	141.7333	6.08	0.001481	N0-N2, N0-N3
	2005		129.7412	3	43.2471	2.00	0.127457	

Notes: FL—fibre length; DCWT—double cell wall thickness; FLD—fibre lumen diameter; FLA—fibre lumen area; VLD—vessel lumen diameter; VLA—vessel lumen area; RA—ray area; CWA—cell wall area; SS—sum of squares; d.f.—degrees of freedom; MS—mean sum of squares. * Post hoc: significant difference in pairs of treatments between-,/in case of multiple pairs.

**Table 4:** Results of repeated measures analysis of variance ANOVA for the anatomical properties of the juvenile wood of the clone ‘Villafranca’ in two years (2006 and 2009) after nitrogen fertilisation.

Property	Year	Source	SS	d.f.	MS	F	*p*	Tukey Post Hoc *
FL (mm)	2006	Treatment	0.6389	3	0.2130	14.24	0.000000	N0-N3, N1-N2, N1-N3
	2009		0.1379	3	0.0460	2.38	0.068249	
DCWT (µm)	2006	Treatment	26.9698	3	8.9899	14.27	0.000000	N0-N1, N1-N2, N1-N3
	2009		9.8136	3	3.2712	5.34	0.001264	N0-N1, N0-N3
FLD (µm)	2006	Treatment	56.0112	3	18.6704	2.80	0.045806	N0-N3
	2009		7.5686	3	2.5229	0.69	0.563483	
FLA (%)	2006	Treatment	398.5898	3	132.8633	5.24	0.003526	N1-N3, N2-N3
	2009		255.3311	3	85.1104	3.50	0.023118	N1-N3
VLD (µm)	2006	Treatment	459.5141	3	153.1714	1.24	0.300380	
	2009		644.9934	3	214.9978	2.37	0.077259	
VLA (%)	2006	Treatment	31.9503	3	10.6501	0.45	0.718302	
	2009		58.1498	3	19.3833	1.42	0.242953	
RA (%)	2006	Treatment	23.4016	3	7.8005	2.90	0.045570	N1-N3
	2009		13.1649	3	4.3883	2.25	0.095309	
CWA (%)	2006	Treatment	257.6216	3	85.8739	2.64	0.061452	
	2009		548.7042	3	182.9014	9.03	0.000089	N0-N3, N1-N3, N2-N3

Notes: FL—fibre length; DCWT—double cell wall thickness; FLD—fibre lumen diameter; FLA—fibre lumen area; VLD—vessel lumen diameter; VLA—vessel lumen area; RA—ray area; CWA—cell wall area; SS—sum of squares; d.f.—degrees of freedom; MS—mean sum of squares. * Post hoc: significant difference in pairs of treatments between -.

**Table 5:** Descriptive statistics for the juvenile wood annual ring widths (ARW) of the clone ‘Villafranca’ in different years.

Property	Year	Treatment									
		Σ		N0		N1		N2		N3	
		Mean	SD	Mean	SD	Mean	SD	Mean	SD	Mean	SD
ARW (mm)	2004	20.72	0.92	20.17	0.85	21.97	5.00	19.91	5.20	20.83	4.18
	2005	22.23	0.38	21.86	2.27	22.76	1.65	22.20	2.43	22.11	2.24
	2006	14.87	0.71	14.04	2.07	15.34	3.05	15.57	1.03	14.53	3.07
	2009	8.99	0.75	8.76	2.88	9.37	2.84	9.77	1.74	8.05	2.15

Notes: ARW—annual ring width; SD—standard deviation.

**Table 6:** Correlation coefficients of the annual ring width and anatomical properties of the juvenile wood of the clone ‘Villafranca’ during and after fertilisation.

	Correlation Coefficient							
Treatment	FL-ARW	DCWT-ARW	FLD-ARW	FLA-ARW	VLD-ARW	VLA-ARW	RA-ARW	CWA-ARW
N0	−0.9573	−0.9380	0.5023	0.9669	−0.7904	−0.8504	0.9844	−0.5635
N1	−0.9514	−0.1085	−0.4026	0.1382	−0.8819	−0.9837	0.9190	0.7156
N2	−0.8856	−0.2639	−0.6483	0.4477	−0.9266	−0.9642	0.9861	0.6840
N3	−0.9411	−0.9844	−0.5027	0.9963	−0.8504	−0.9518	0.9513	−0.7553
ALL	−0.9176	−0.3092	−0.3330	0.6023	−0.8437	−0.9456	0.9152	0.1227

Notes: ARW—annual ring width; FL—fibre length; FLD—fibre lumen diameter; FLA—fibre lumen area; VLD—vessel lumen diameter; VLA—vessel lumen area; RA—ray area; CWA—cell wall area.

## Data Availability

The data presented in this study are available upon request from the corresponding author.

## References

[1] Richardson J., Isebrands J.G., Ball J.B., Isebrands J.G., Richardson J. (2014). Ecology and physiology of poplars and willows. Poplars and Willows: Trees for Society and the Environment.

[2] Dickmann D.I., Kuzovkina J., Isebrands J.G., Richardson J. (2014). Poplars and willows in the world, with emphasis on silviculturally important species. Poplars and Willows: Trees for Society and the Environment.

[3] Šijačić-Nikolić M. (2019). Variability of white poplar clones in a nursery trial. Bull. Fac. For..

[4] Poljaković-Pajnik L., Drekić M., Kovačević B., Milović M., Novčić Z., Vasić V. (2020). Predilection of Chaitophorus populeti and Phyllobius oblongus on four clones of white poplar. Topola.

[5] Schenone G., Facciotto G., Groppi F., Mughini G., Pari L. (2000). *Short Rotation Woody Crops for Energy: The Research Program of ENEL (Italian Electric Company)*; 1997. In: Confalonieri, M.; Belenghi, B.; Balestrazzi, A.; Negri, S.; Facciotto, G.; Schenone, G.; Delledonne, M. Transformation of elite white poplar (*Populus alba* L.) cv. ‘Villafranca’ and evaluation of herbicide resistance. Plant Cell Rep..

[6] Rosso L., Facciotto G., Bergante S., Vietto L., Nervo G. (2013). Selection and testing of *Populus alba* and *Salix* spp. as bioenergy feedstock: Preliminary results. Appl. Energy.

[7] Pelleri F., Ravagni S., Bianchetto E., Bidini C. (2013). Comparing growth rate in a mixed plantation (walnut, poplar and nurse trees) with different planting designs: Results from an experimental plantation in northern Italy. Ann. Silvic. Res..

[8] Benko M., Malez V., Kranjc Z. Kuda idu dravske šume?. Proceedings of the Symposium, Zaštita Prirode i Okoliša i Eksploatacija Mineralnih Sirovina, Hrvatsko Ekološko Društvo—Ekološke Monografije.

[9] Evans J., Turnbull J. (2004). Plantation Forestry in the Tropics.

[10] Hart J.F. (2010). A review of the effects of silviculture on wood quality. WOOD 493.

[11] Smethurst P.J. Forest fertilisation: Trends in knowledge and practice compared to agriculture. Proceedings of the IPI-OUAT-IPNI, International Symposium, Bhubaneswar.

[12] Thevathasan N.V., Reynolds P.E., Kuessner R., Bell W.F. (2000). Effects of controlled weed densities and soil types on soil nitrate accumulation, spruce growth, and weed growth. For. Ecol. Manag..

[13] Van den Driessche R., Rude W., Martens L. (2003). Effect of fertilisation and irrigation on growth of aspen (Populus tremuloides Michx.) seedlings over three seasons. For. Ecol. Manag..

[14] Singh B., Behl H.M. (1999). Energy flow, carbon and nitrogen cycling in Populus deltoides clones in north India. Biomass Bioenergy.

[15] Adegbidi H.G., Volk T.A., White E.H., Abrahamson L.P., Briggs R.D., Bickelhaupt D.H. (2001). Biomass and nutrient removal by willow clones in experimental bioenergy plantations in New York State. Biomass Bioenergy.

[16] Waring R.H., Youngberg C.T. (1972). Evaluating forest sites for potential growth response of trees to fertiliser. Northwest Sci..

[17] Stanturf J.A., van Oosten C., Eds Isebrands J.G., Richardson J. (2014). Operational poplar and willow culture. Poplars and Willows: Trees for Society and the Environment.

[18] Zobel B.J. (1992). Silvicultural effects on wood properties. IPEF Int. Piracicaba.

[19] Stettler R.F., Bradshaw H.D., Heilman P.E., Hinckley T.M. (1996). Biology of Populus and Its Implications for Management and Conservation.

[20] Zobel B.J., van Buijetenen J.P. (1989). Wood Variation. Its Causes and Control.

[21] Ferm A., Hytönen J., Vuori J. (1989). Effect of spacing and nitrogen fertilisation on the establishment and biomass production of short rotation poplar in England. Biomass.

[22] Misra P.N., Tewari S.K. (1999). On the performance of poplars (*Populus deltoides*) on marginal soils in northern India. Biomass Bioenergy.

[23] Bilodeau-Gauthier S., Paré D., Messier C., Bélanger N. (2011). Juvenile growth of hybrid poplars on acidic boreal soil determined by environmental effects of soil preparation, vegetation control, and fertilisation. For. Ecol. Manag..

[24] Khamis M.H., Atia M.G., Ali H.M. (2013). Impact of nitrogen and phosphorus sources on growth efficiency of Melia Azedarch and Populus Euphratica in Wadi El Natrun, Egypt. J. For. Ind..

[25] From F., Strengbom J., Nordin A. (2015). Residual long-term effects of forest fertilisation on tree growth and nitrogen turnover in boreal forest. Forests.

[26] Panshin A.J., De Zeeuw C. (1980). Textbook of Wood Technology: Structure, Identification, Properties, and Uses of the Commercial Woods of the United States and Canada.

[27] DeBell D.S., Mallonee E.H., Alford L.T. (1975). Effect of nitrogen fertiliser on growth, form, and wood quality of Eastern Cottonwood. For. Res. Pap..

[28] Luo Z.B., Langenfeld-Heyser R., Calfapietra C., Polle A. (2005). Influence of free air CO_2_ enrichment (EUROFACE) and nitrogen fertilisation on the anatomy of juvenile wood of three poplar species after coppicing. Trees.

[29] Li H., Li M., Luo J., Cao X., Qu L., Gai Y., Jiang X., Liu T., Bai H., Janz D. (2012). N-fertilization has different effects on the growth, carbon and nitrogen physiology, and wood properties of slow- and fast-growing *Populus* species. J. Exp. Bot..

[30] Hacke U.G., Plavcova L., Almeida-Rodriguez A., King-Jones S., Zhou W., Cooke J.E.K. (2010). Influence of nitrogen fertilisation on xylem traits and aquaporin expression in stems of hybrid poplar. Tree Physiol..

[31] Ištok I., Šefc B., Hasan M., Popović G., Sedlar T. (2017). Fiber characteristics of white poplar (*Populus alba* L.) juvenile wood along the Drava river. Drv. Ind..

[32] CMHS Croatian Meteorological and Hydrological Service. https://meteo.hr/klima.php?section=klima_podaci&param=k1&Grad=varazdin.

[33] Franklin G.L. (1945). Preparation of thin-wood sections of synthetic resins and wood-resin composites, and a new macerating method for wood. Nature.

[34] Peszlen I. (1994). Influence of age on selected anatomical properties of populus clones. IAWA J..

[35] Sicard C., Saint-Andre L., Gelhaye D., Rangger J. (2006). Effect of initial fertilisation on biomass and nutrient content of Norway spruce and Douglas-fir plantations at the same site. Trees.

[36] Nyakuengama J.G., Downes G.M., Ng J. (2002). Growth and wood density response to large-age fertiliser application in Pinus radiata. IAWA J..

[37] Little K.M., Johannes van Standen G., Clarke P.Y. (2003). The relationship between vegetation management and the wood and pulping properties of a Eucalyptus hybrid clone. Ann. For. Sci..

[38] Pitre F.E., Cooke J.E.K., Mackay J.J. (2007). Short-term effects on nitrogen availability on wood formation and fibre properties in hybrid poplar. Trees.

[39] Wodzicki T.J. (2001). Natural factors affecting wood structure. Wood Sci. Technol..

[40] Monteoliva S.E., Villegas M.S., Achinelli F.G. (2015). Short-term and long-term effects of weed control and fertilisation on growth and wood anatomy of a Populus deltoides clone. For. Syst..

[41] Raymond C.A., Muneri A. (2000). Effect of fertiliser on wood properties of Eucalyptus globulus. Can. J. For. Res..

[42] Campion J.M. (2009). The effects of mid- and late- rotation fertiliser application on tree growth and wood quality in softwood saw-timber stands: A critical review. South. For..

[43] Tsoumis G. (1991). Science and Technology of Wood: Structure, Properties, Utilisation.

[44] Zobel B.J., Sprague J.R. (1998). Juvenile Wood in Forest Trees.

[45] Bendtsen B.A. (1978). Properties of wood from improved and intensively managed trees. For. Prod. J..

[46] Jeong G.Y., Zink-Sharp A. (2012). Technical note: The impact of fertilisation on within-tree variability in young loblolly pine (*Pinus taeda*). Wood Fiber Sci..

[47] Lundgren C. (2004). Cell wall thickness and tangential and radial cell diameter of fertilised and irrigated Norway spruce. Silva Fenn..

[48] de Lima I.L., Longui E.L., Junior L.S., Garcia J.N., Borges Florsheim S.M. (2010). Effect of fertilisation on cell size in wood of Eucalyptus grandis Hill Ex Maiden. Cerne.

[49] Smolander A., Henttonen H.M., Nöjd P., Soronen P., Mäkinen H. (2022). Long-term response of soil and stem wood properties to repeated nitrogen fertilisation in a N-limited Scots pine stand. Eur. J. For. Res..

[50] Finto A., Schimleck L.R., Daniels R.F., Clark A. (2011). Effect of fertilisation on growth and wood properties of thinned and unthinned midrotation loblolly pine (*Pinus taeda* L.) Stands. South. J. Appl. For..

[51] Balatinecz J.J., Kretschmann D.E., Leclercq A. (2001). Achievements in the utilisation of poplar wood—Guideposts for the future. For. Chron..

[52] Nilsson J.A., Häkansson C., Blom Ä., Bergh J. (2021). Effects of fertilisation on wood formation in naturally regenerated juvenile silver birch in a Norway spruce stand in south Sweden. Forests.

